# Anatomy, histology, and histochemistry of the harderian and lacrimal glands in the great spotted woodpecker (*dendrocopos major*)

**DOI:** 10.1007/s11259-026-11477-w

**Published:** 2026-09-16

**Authors:** Joanna Klećkowska-Nawrot, Aleksandra Kroczak-Zdańkowska, Adam Urantówka, Grzegorz Zaniewicz, Shaw Badenhorst, Aleksander Chrószcz, Krzysztof Stegmann, Karolina Barszcz, Dominik Poradowski

**Affiliations:** 1 Department of Biostructure and Animal Physiology, Division of Animal Anatomy, Faculty of Veterinary Medicine, Wrocław University of Environmental and Life Sciences, Kożuchowska 1, Wrocław, 51-631 Poland; 2 Department of Genetics, Faculty of Biology and Animal Science, Wrocław University of Environmental and Life Sciences, Kożuchowska 7, Wrocław, 51-631 Poland; 3 Department of Vertebrate Ecology and Zoology, Faculty of Biology, University of Gdańsk, Wita Stwosza 59, Gdańsk, 80-308 Poland; 4 Evolutionary Studies Institute, University of the Witwatersrand, 1 Jan Smuts Ave, Braamfontein, Johannesburg 2000 South Africa; 5 RZGW, C. K. Norwida 34, Wrocław, 50-950 Poland; 6 Department of Morphological Sciences, Institute of Veterinary Medicine, Warsaw University of Life Sciences, Nowoursynowska 159, Warsaw, 02-787 Poland

**Keywords:** Avian ophthalmology, Ocular adnexa, Harderian gland, Lacrimal gland, Histochemical analysis, Great spotted woodpecker

## Abstract

The Harderian and lacrimal glands are the main orbital glands of the avian lacrimal apparatus and contribute to the tear film, nictitating membrane function, and local protection of the ocular surface. Although the ocular adnexa have been well described in domestic birds, the morphology and function of these organs remain poorly characterized in free-living species. This study aimed to characterize the morphology and histochemistry of these glands in the Great Spotted Woodpecker. The material consisted of 18 individuals found dead in the natural environment in Poland. Gross anatomical, morphometric, histological, and histochemical analyses were performed. Both glands occupied typical orbital positions. The secretory units of the Harderian gland consisted of one predominant epithelial cell type, contrasting with the more heterogeneous epithelial organization reported in some other avian species. Both glands were compound tubuloacinar structures. The Harderian gland opened into the lower conjunctival sac, whereas the smaller lacrimal gland drained into the dorsal conjunctival sac. The Harderian gland had a more homogeneous secretory parenchyma and a well-developed excretory duct system. The overall morphology of both glands was comparable to that described in other birds. However, the histochemical secretory profile differed from that reported in several species: the Harderian gland produced mainly acidic mucosubstances, whereas the lacrimal gland showed a mixed secretory profile. The immune component of both glands had a diffuse and periductal distribution, which contrasted with the pattern described in some birds. These findings show that the Harderian and lacrimal glands are structurally and histochemically distinct and suggest complementary roles in ocular surface maintenance in this species. At the same time, the results indicate selected morphological and functional differences compared with both domestic and other free-living birds.

**Supplementary Information:**

The online version contains supplementary material available at https://doi.org/10.1007/s11259-026-11477-w.

## Introduction

The Harderian gland (*glandula membranae nictitantis*) and lacrimal gland (*glandula lacrimalis*) are the main orbital glands of the avian lacrimal apparatus (Baumel et al. 1993; Payne 1994; Shirama et al. 1996). Together with the eyelids, conjunctiva, and nictitating membrane, they help maintain the avian ocular surface, which is exposed to dust, aerosols, plant particles, and microorganisms, especially in free-ranging birds. For this reason, the structure and secretion of the orbital glands are relevant to the condition of the conjunctival sac, nictitating membrane, and precorneal tear film.

In birds, the Harderian gland is usually the largest orbital gland. Its excretory duct opens into the conjunctival space between the nictitating membrane and the eyeball. The secretion of this gland contributes to lubrication of the nictitating membrane and maintenance of the precorneal tear film (Payne 1994; Shirama et al. 1996; Klećkowska-Nawrot et al. 2015a, b, 2016a, b). The gland is also associated with local mucosal immunity because it is exposed to particles and microorganisms (Davison et al. 2008). Previous studies have described organized lymphoid follicles, scattered lymphocytes, plasma cells, and immunoglobulins in the avian Harderian gland and in its secretions (Wight et al. 1971a; Gallego et al. 1992; Payne 1994; Davison et al. 2008; ). Experimental studies have also shown local immune responses after ocular antigen exposure (Gallego et al. 1992). Moreover, the microscopic structure of the avian Harderian gland varies among species and has been classified as compound tubuloacinar, tubular, or mixed (Burns 1974, 1992). The secretory units are usually lined by simple columnar epithelium and may be associated with myoepithelial cells. Plasma cells are often present in the interstitial tissue, but their number and distribution vary among species and age groups (Burns 1992; Shirama et al. 1996; Altunay and Kozlu 2004; Khan et al. 2007; Boydak and Aydin 2009). This variation should be considered when comparing the glandular structure of domestic birds with that of wild species.

The lacrimal gland is generally smaller and has been described as structurally simpler in comparative studies (Burns 1976, 1979; Klećkowska-Nawrot et al. 2015c). It is usually associated with the aqueous component of the tear film, whereas the Harderian gland is more closely related to the nictitating membrane and conjunctival surface (Walcott and McLean 1985; Payne 1994; Walcott 1998). However, both glands are part of the avian lacrimal apparatus and should be considered together when describing glandular structures of the orbit.

Earlier histochemical studies have shown that the secretory profiles of the Harderian and lacrimal glands differ among bird species. Acidic and neutral mucins, sulfated and carboxylated mucosubstances, and glycoprotein-rich secretions have been reported in different proportions in the glandular epithelium and duct system (Dimitrov and Nikiforov 2005; Mobini 2012; Beheiry et al. 2020). Such variation may be related to differences in tear film composition, lubrication of the nictitating membrane, and protection of the conjunctival and corneal surfaces. Most available data, however, concern domestic birds or selected nondomestic species. Therefore, detailed histochemical characterization of the lacrimal apparatus glands in free-living birds is important for further comparative studies.

The Great Spotted Woodpecker (*Dendrocopos major*) is a common and widespread representative of Picidae in Eurasia and North Africa (Winkler et al. 2020). However, detailed data on the gross anatomy, microscopic organization, and histochemical profile of its Harderian and lacrimal glands are lacking. Therefore, this study aimed to fill this gap and provide baseline morphological information for future studies of avian ocular adnexa.

## Materials and methods

### Animals and sample collection

The study was performed on 18 Great Spotted Woodpeckers (*D. major*) obtained postmortem between 2020 and 2023 in Wrocław, Poland. The sample included seven females and 11 males. Specimens were collected from wooded habitats, including forests and parkland (Table 1). No birds were killed, captured, or handled for this study. After collection, samples were stored at approximately − 20 °C, and tissue preservation was evaluated during dissection and preliminary histological examination. Individuals were classified as juvenile, immature, or adult based on general plumage characteristics, according to published criteria (Baker 2016; Blasco-Zumeta and Heinze 2026; Jenni et al. 2026). Because sex determination based on morphology and gonadal examination was not always possible, particularly in juveniles, final sex identification was performed using molecular methods.

### Molecular sex determination of the analyzed individuals

No published study has yet reported the use of CHD1-based molecular sexing in the Great Spotted Woodpecker. Therefore, we assessed whether the molecular marker commonly used for avian sex identification, based on length polymorphism in intron 16 of the CHD1 gene, was suitable for sex determination in this species (Fridolfsson and Ellegren 1999; Kroczak et al. 2021). To validate marker effectiveness, PCR reactions were initially performed for one male and one female of confirmed sex, as determined from genital apparatus morphology. DNA fragments were amplified by preparing the reaction mixture according to Kroczak et al. (2021) and using the CHD1iA amplification protocol (Kroczak et al. 2021). After it was confirmed that the molecular marker enabled unambiguous sex identification based on an easily interpretable banding pattern of PCR products obtained from males and females, the PCR-based sexing assay was applied to all individuals included in the study. The results are presented in Supplementary Figure S1. Information on sex and age class for all studied individuals is presented in Table 1.

### DNA extraction

Total DNA was extracted from breast feathers using the Sherlock AX Kit (A&A Biotechnology, Gdynia, Poland) according to the manufacturer’s protocol.

### Electrophoretic separation of amplified DNA fragments

PCR products were separated by electrophoresis on a 1% agarose gel. The gel contained the fluorescent dye Syngen GreenDNA Gel Stain (Syngen Biotech Sp. z o.o.), added at a volume of 0.8 µL per 120 mL of dissolved agarose. The procedure was carried out in 1× TAE buffer at a voltage of 2–5 V/cm. DNA fragment sizes were assessed using the GeneRuler 100 bp Plus DNA Ladder (Thermo Scientific), recommended for determining the size of double-stranded DNA fragments ranging from 100 bp to 3000 bp in agarose gels. The electrophoretic separation results were visualized under ultraviolet light using a UVP GelDoc-It2 Imaging System (Analytik Jena).

### Weighing

Body mass was measured before eye dissection and carcass freezing using a WPE 300 electronic balance (Radwag, Poland; readability 10 mg). Body mass data for all individuals are presented in Table 1.

**Table 1:** Characteristics of the Great Spotted Woodpeckers (*Dendrocopos major*) examined in the study. Records are arranged by age, sex, and descending body mass (g)

Individual no.	Body mass (g)	Age	Sex
1	63.61	juvenile	female
2	67.18	juvenile	male
3	63.33	adult	male
4	50.95	adult	male
5	81.10	adult	female
6	79.93	adult	male
7	57.53	adult	male
8	60.99	adult	male
9	52.80	juvenile	male
10	55.67	juvenile	female
11	62.89	adult	male
12	55.46	adult	male
13	68.46	juvenile	female
14	72.00	immature	female
15	57.00	juvenile	male
16	33.74	juvenile	female
17	45.00	juvenile	female
18	73.45	adult	male

### Macroscopic examination and morphometry

Both orbital regions were dissected from each bird, and both glands were collected bilaterally. During dissection, the eyeball and associated orbital glands were examined under a Zeiss Stemi 2000-C stereomicroscope (Carl Zeiss, Jena, Germany). The position of each gland within the orbit, its relationship to adjacent orbital structures, and its shape and color were described. Morphometric measurements were performed only on glands in which the external contour, shape, and anatomical boundaries were clearly preserved. The length and width of the organs were measured using Axio Vision Release 4.8.2 SP2 software (Carl Zeiss MicroImaging GmbH, Jena, Germany). The mean value of each parameter was calculated. Morphometric data were summarized using descriptive statistics, including the mean, standard deviation, minimum, and maximum values. No inferential statistical comparisons were performed because of the descriptive nature of the study. Images were acquired using the same stereomicroscope equipped with a Canon EOS 300D digital camera (Canon Inc., Tokyo, Japan).

#### Ethical statement

The study was conducted in accordance with Polish and European regulations. All material originated from birds found dead in the natural environment; therefore, approval from a Local Ethics Committee for Animal Experimentation was not required under Polish law or Directive 2010/63/EU. Collection, possession, transfer, and storage of the material were carried out under permits issued by the Regional Directors for Environmental Protection in Wrocław (WPN.6401.83.2021.MH) and Gdańsk (WZG.6401.248.2020.AN.2), and by the District Veterinary Officer in Wrocław (PU.555.15.2020). Copies of permits are available from the corresponding author on reasonable request.

#### Tissue processing and paraffin embedding

Tissue samples were fixed in 4% buffered formaldehyde for 72 h and then rinsed in running tap water for 24 h. The material was dehydrated in a graded ethanol series of 75%, 96%, and 100% using an ETP RVG3 vacuum tissue processor (Intelsint, Italy), cleared in xylene, and embedded in paraffin. Serial 4-µm-thick sections were cut using a Slide 2003 sliding microtome (Pfm A.G., Germany). The stained sections were examined using a Zeiss Axio Scope A1 light microscope (Carl Zeiss, Jena, Germany), and images were acquired with Axio Vision Release 4.8.2 SP2 software (Carl Zeiss MicroImaging GmbH, Jena, Germany). Anatomical and histological terminology followed the Nomina Anatomica Avium (Baumel et al. 1993) and the Nomina Histologica Veterinaria (2017), where applicable. Histological images were adjusted for brightness and contrast using the Fiji distribution of ImageJ software (version 1.54p; National Institutes of Health, Bethesda, MD, USA; Schindelin et al. 2012). No changes were made that altered the original histological information.

#### Histological and histochemical staining

Histological and histochemical staining was performed to characterize the general structure, connective tissue framework, innervation, plasma cell distribution, and secretory profile of the Harderian and lacrimal glands. The staining procedures were performed according to the manufacturers’ protocols and are described in detail in the Supplementary Material (Table S1). The intensity of histochemical reactions was evaluated semiquantitatively according to a scale modified from Spicer and Henson (1967): negative reaction, −; weak reaction, −/+ to +; moderate reaction, ++ to ++/+++; and strong reaction, +++. The evaluation considered both staining intensity and the distribution of positive reactions within the secretory epithelium and ductal system.

## Results

Mayer’s hematoxylin and eosin staining was used to evaluate glandular tissue preservation and to select samples suitable for further histological and histochemical analyses. This preliminary screening showed early postmortem autolytic changes in the glandular tissue of juvenile birds. Although these changes did not prevent macroscopic examination and morphometry, juvenile glandular samples were excluded from the final histological and histochemical analyses.

### Macroscopic morphology and morphometry of the harderian and lacrimal glands

**Fig. 1:**
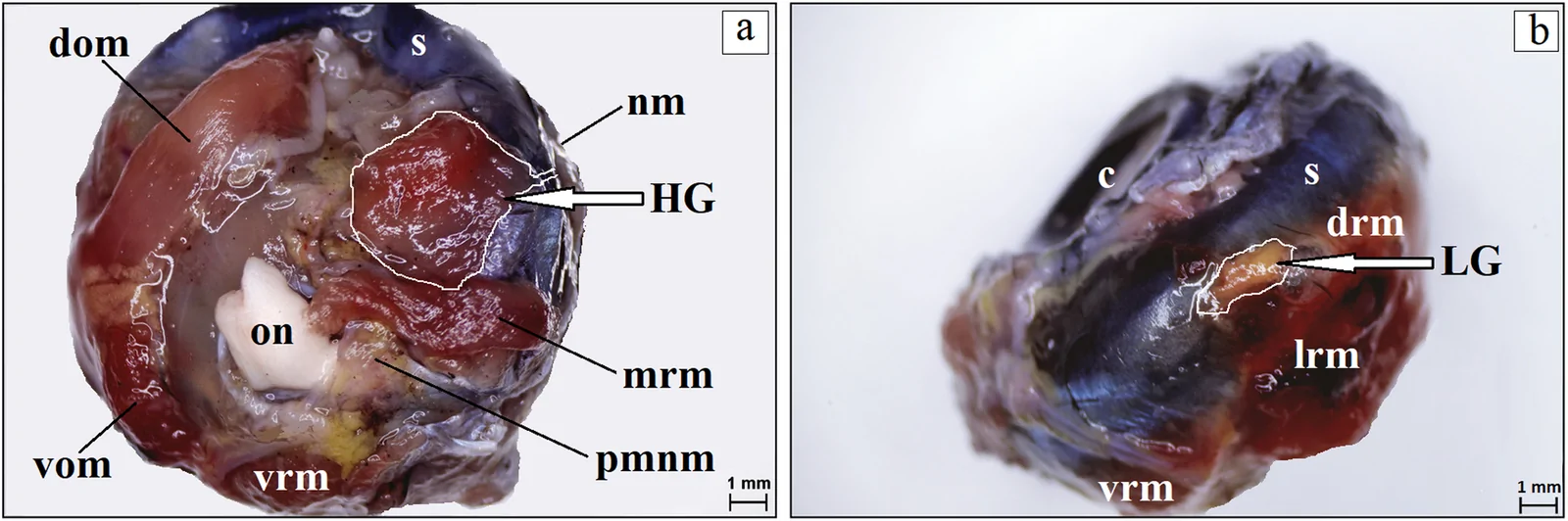
Macroscopic appearance and topographic relationships of the Harderian and lacrimal glands in the orbital region of the Great Spotted Woodpecker (*Dendrocopos major*). (**a**) Dissected orbital region showing the Harderian gland (HG) and its relationship to the optic nerve and extraocular muscles. (**b**) Dissected orbital region showing the lacrimal gland (LG) in relation to the cornea, sclera, and extraocular muscles. The glandular tissue is outlined in white. Abbreviations: c, cornea; dom, dorsal oblique muscle; drm, dorsal rectus muscle; HG, Harderian gland; LG, lacrimal gland; lrm, lateral rectus muscle; mrm, medial rectus muscle; nm, nictitating membrane; on, optic nerve; pmnm, pyramidal muscle of the nictitating membrane; s, sclera; vom, ventral oblique muscle; vrm, ventral rectus muscle. Scale bars: 1 mm

The Harderian gland of the Great Spotted Woodpecker was located ventromedially in the orbit, close to the interorbital septum, between the medial rectus muscle, the pyramidal muscle of the nictitating membrane, and the ventral oblique muscle. The gland was oval and light pink (Fig. 1a). A single excretory duct was observed opening into the lower conjunctival sac between the nictitating membrane and the cornea. The lacrimal gland was located dorsolaterally in the orbit, between the lateral rectus and dorsal rectus muscles, close to the pyramidal muscle of the nictitating membrane and its tendon. The gland was elongated, flattened, undivided, and light pink (Fig. 1b). Lacrimal secretion was drained by multiple excretory ducts opening into the dorsal conjunctival fornix.

**Fig. 2:**
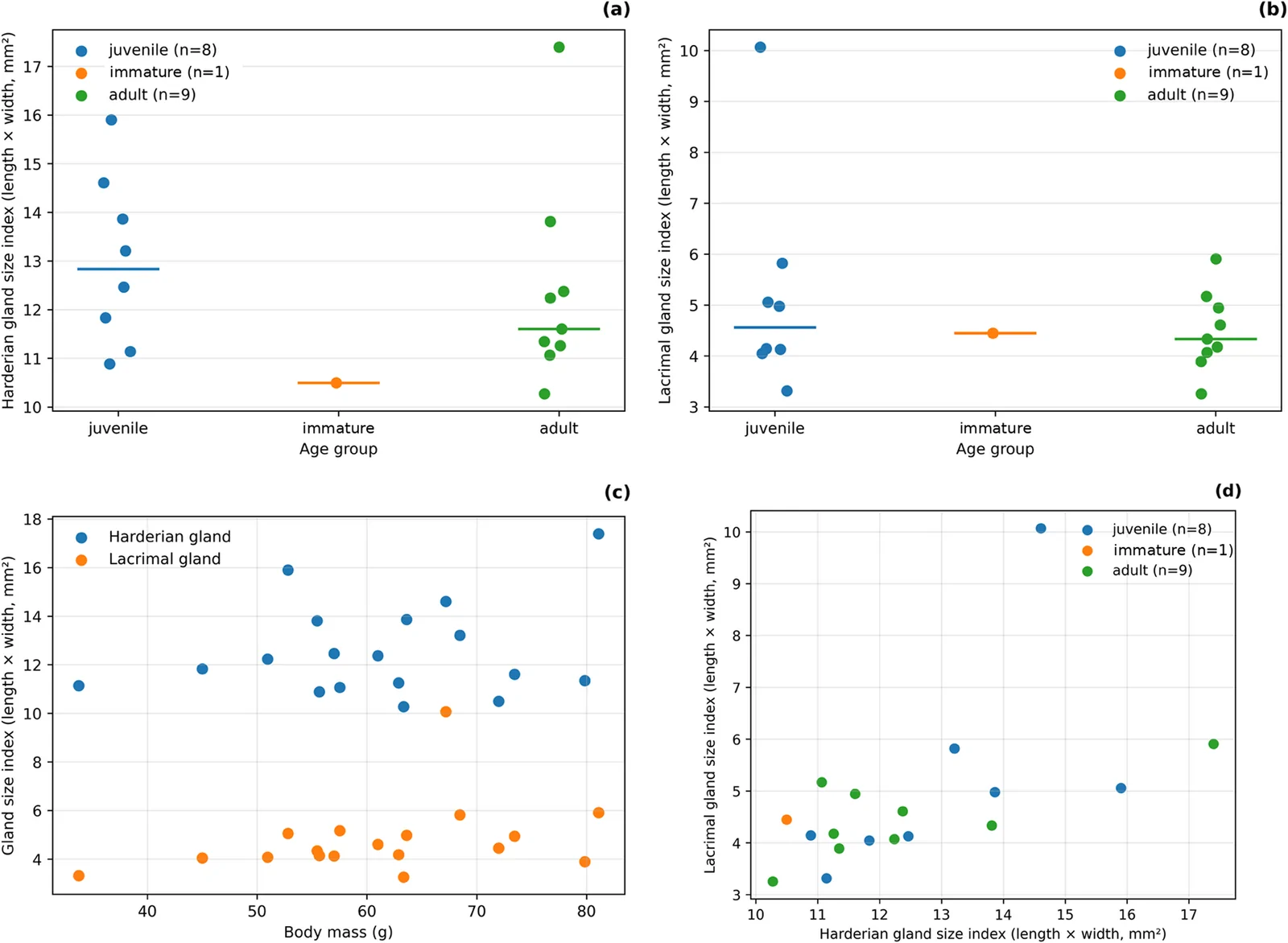
Morphometric relationships of the Harderian and lacrimal glands in the Great Spotted Woodpecker. (**a**) Harderian gland size index by age group. (**b**) Lacrimal gland size index by age group. (**c**) Relationship between body mass and gland size indices. (**d**) Relationship between Harderian and lacrimal gland size indices. The gland size index was calculated as length × width (mm²). In panels (**a**) and (**b**), colors indicate age group and horizontal lines indicate median values; in panel (**c**), colors indicate gland type; in panel (**d**), colors indicate age group

Detailed descriptive statistics for the entire sample are presented in Table 2. The gland size index, calculated as gland length × width, showed that the Harderian gland had a higher size index than the lacrimal gland, with a mean Harderian gland/lacrimal gland index ratio of 2.72. Descriptive comparison by age group did not reveal a clear age-related pattern in gland size indices (Table 3, Fig. 2a, b). The immature category was represented by only one individual; therefore, this value should be interpreted descriptively only. Descriptive comparison by sex also did not reveal an obvious sex-related pattern in the measured parameters (Table 3). The differences between males and females were small, and the value ranges partially overlapped between the two groups. The distribution of individual values showed moderate interindividual variation, which was more apparent for the lacrimal gland than for the Harderian gland. This was particularly evident in juvenile birds, which showed the widest range of lacrimal gland size index values. Scatterplots did not show a clear relationship between body mass and gland size indices (Fig. 2c). No consistent pattern was observed between the Harderian gland size index and the lacrimal gland size index (Fig. 2d). The graphs are intended to illustrate the distribution of individual descriptive values rather than to indicate statistically tested age- or sex-related differences.

**Table 2:** Descriptive statistics for body mass and morphometric parameters of the Harderian and lacrimal glands in the examined Great Spotted Woodpeckers (*Dendrocopos major*; *n* = 18). Data are presented as sample size (n), mean, standard deviation (SD), median, minimum, and maximum

Variable	Mean	SD	Median	Min	Max
**Body mass (g)** (*n* = 18)	61.17	11.88	61.94	33.74	81.1
**HG length (mm)** (*n* = 18)	5.04	0.47	4.97	4.48	6.49
**HG width (mm)** (*n* = 18)	2.49	0.29	2.41	2.14	3.14
**HG size index (mm²)** (*n* = 18)	12.54	1.94	12.03	10.27	17.4
**LG length (mm)** (*n* = 18)	2.37	0.37	2.26	1.95	3.52
**LG width (mm)** (*n* = 18)	1.99	0.28	1.92	1.57	2.86
**LG size index (mm²)** (*n* = 18)	4.80	1.5	4.39	3.26	10.07
**HG/LG index ratio** (*n* = 18)	2.72	0.47	2.85	1.45	3.36

**Table 3:** Descriptive statistics for body mass and morphometric parameters of the Harderian and lacrimal glands in the examined great spotted woodpeckers (*Dendrocopos major*) by age class and sex. Data are presented as mean ± SD. Because only one sub-adult individual was available, this category is presented as a single value

Age group	Body mass (g)	HG length (mm)	HG width (mm)	HG size index (mm²)	LG length (mm)	LG width (mm)	LG size index (mm²)
**Juvenile** (*n* = 8)	55.43 ± 11.73	5.06 ± 0.63	2.57 ± 0.25	12.99 ± 1.75	2.45 ± 0.47	2.06 ± 0.39	5.19 ± 2.11
**Immature** (*n* = 1)	72.00	4.49	2.33	10.5	2.27	1.96	4.45
**Adult** (*n* = 9)	65.06 ± 10.71	5.09 ± 0.29	2.42 ± 0.34	12.37 ± 2.13	2.31 ± 0.29	1.93 ± 0.14	4.48 ± 0.78
**Female** (*n* = 7)	59.94 ± 16.37	4.83 ± 0.39	2.61 ± 0.35	12.69 ± 2.41	2.38 ± 0.25	1.95 ± 0.23	4.67 ± 0.96
**Male** (*n* = 11)	61.95 ± 8.8	5.18 ± 0.49	2.40 ± 0.24	12.45 ± 1.69	2.36 ± 0.44	2.02 ± 0.31	4.88 ± 1.81

### Histological and histochemical characteristics of the harderian gland

**Fig. 3:**
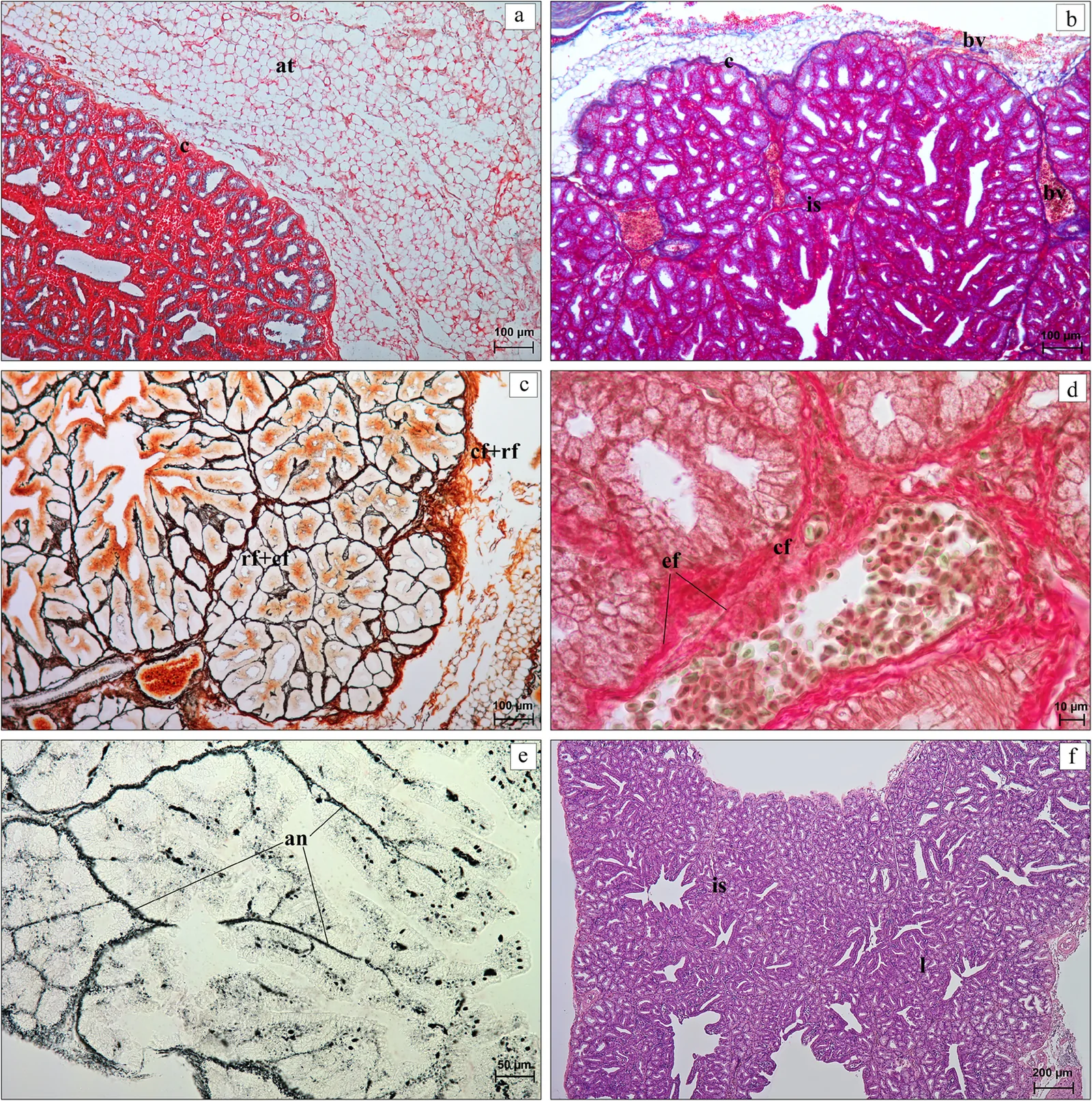
Histological organization of the Harderian gland in the Great Spotted Woodpecker. Representative micrographs from well-preserved adult and subadult specimens show a multilobular gland surrounded by a distinct connective tissue capsule and abundant orbital adipose tissue. The glandular parenchyma is divided into lobules by interlobular septa, which contain blood vessels and connective tissue fibers. Movat pentachrome and Heidenhain’s Azan trichrome staining demonstrate the collagen-rich capsule and septa, whereas reticulin staining shows a delicate network of reticular fibers supporting the glandular lobules. Verhoeff staining highlights elastic fibers, mainly within the stromal and perivascular connective tissue. Bielschowsky staining demonstrates nerve fibers/axonal elements within the glandular stroma. Hematoxylin and eosin staining confirms the compact lobular arrangement of the gland and the distribution of interlobular connective tissue. Abbreviations: an, axonal elements/nerve fibers; at, adipose tissue; bv, blood vessel; c, capsule; cf, collagen fibers; ef, elastic fibers; is, interlobular septum; l, lobule; rf, reticular fibers. (**a**) Movat pentachrome (modified Russell-Movat); (**b**) Heidenhain’s Azan trichrome; (**c**) reticulin; (**d**) Verhoeff; (**e**) Bielschowsky; (**f**) Mayer’s hematoxylin and eosin. Scale bars: (**a**–**c**) 100 µm; (**d**) 10 µm; (**e**) 50 µm; (**f**) 200 µm.

**Fig. 4:**
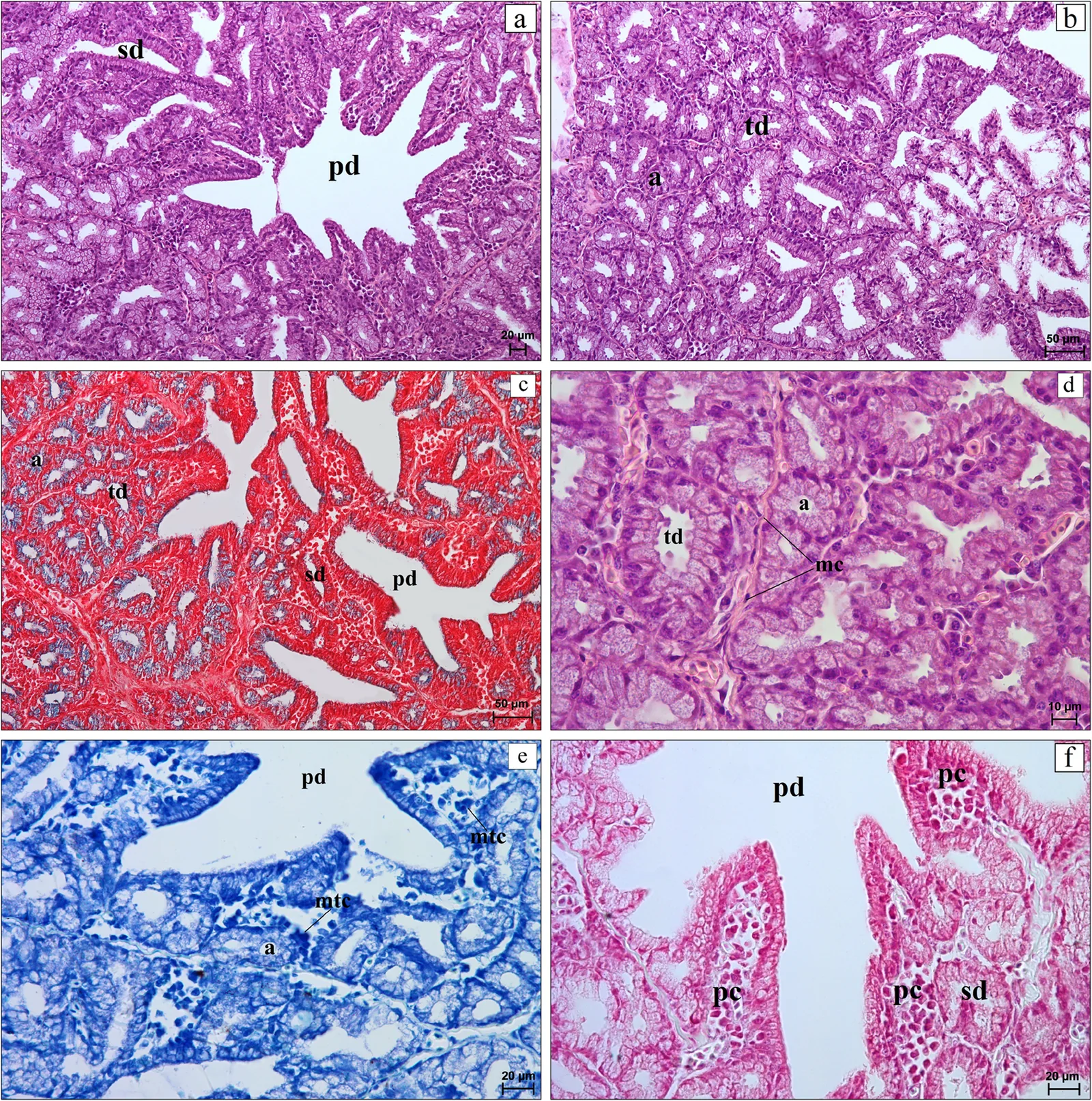
Ductal and secretory organization of the Harderian gland in the Great Spotted Woodpecker. Representative micrographs from adult and subadult specimens show a well-developed ductal system surrounded by secretory acini. The primary duct is characterized by a wide, irregular lumen and is accompanied by secondary and tertiary ducts that branch within the glandular parenchyma. Secretory acini are closely arranged around the ductal profiles, and flattened myoepithelial cells are visible at the basal surface of the acini and small ducts. Movat pentachrome staining also shows the connective tissue framework surrounding the ducts and acini. Toluidine blue polychrome staining demonstrates metachromatic mast cells within the glandular stroma, whereas methyl green–pyronin staining highlights periductal plasma cells, particularly around the larger ducts. Abbreviations: a, acini; mc, myoepithelial cells; mtc, metachromatic mast cells; pc, plasma cells; pd, primary duct; sd, secondary duct; td, tertiary duct. (**a**, **b**, **d**) Mayer’s hematoxylin and eosin; (**c**) Movat pentachrome (modified Russell-Movat); (**e**) toluidine blue polychrome; (**f**) methyl green–pyronin. Scale bars: (**a**, **e**, f) 20 µm; (**b**, **c**) 50 µm; (**d**) 10 µm

In the nonjuvenile material examined, the Harderian gland was enclosed by a thin connective tissue capsule and was externally surrounded by adipose tissue and numerous blood vessels (Figs. 3a, b). The capsule was composed mainly of dense collagenous connective tissue, with reticular fibers and a small number of elastic fibers (Figs. 3c, d). The interlobular septa contained blood vessels and nerve fibers and were composed predominantly of reticular and collagen fibers, with only weakly developed elastic fibers (Figs. 3c–e). Thin connective tissue septa extended from the capsule into the glandular parenchyma and divided it into lobules of variable size (Fig. 3f). The Harderian gland showed a Type I compound tubuloacinar organization (Fig. 3f). The glandular parenchyma consisted of one type of secretory lobule and one predominant type of secretory epithelial cell (Figs. 4a, c). A large, irregular, and often branched primary ductal system was present in the central region of each lobule (Fig. 4a). The primary ducts were lined by tall columnar epithelial cells with large, oval, basally located nuclei (Figs. 4a, b). Secondary and tertiary ducts had smaller, oval to elongated lumina and were lined by a single layer of cuboidal epithelial cells (Figs. 4a, b). The secretory acini were located mainly in the peripheral part of the lobules and were connected with tertiary and secondary ducts. The acini consisted of pyramidal secretory cells with slightly granular, eosinophilic cytoplasm and narrow lumina (Fig. 4d). Myoepithelial cells were observed around the secretory acini (Fig. 4d). Toluidine blue staining revealed metachromatic mast cells scattered within the interlobular connective tissue, especially near blood vessels and ducts (Fig. 4e). Plasma cells and lymphocytes were present in small numbers in the peripheral lobular interstitium, interacinar connective tissue, and connective tissue between secondary and tertiary ducts (Fig. 4f). These cells were more numerous around the primary ducts. Plasma cells showed an eccentric nucleus and pink to pink-red cytoplasm in methyl green–pyronin-stained sections (Fig. 4f).

**Fig. 5:**
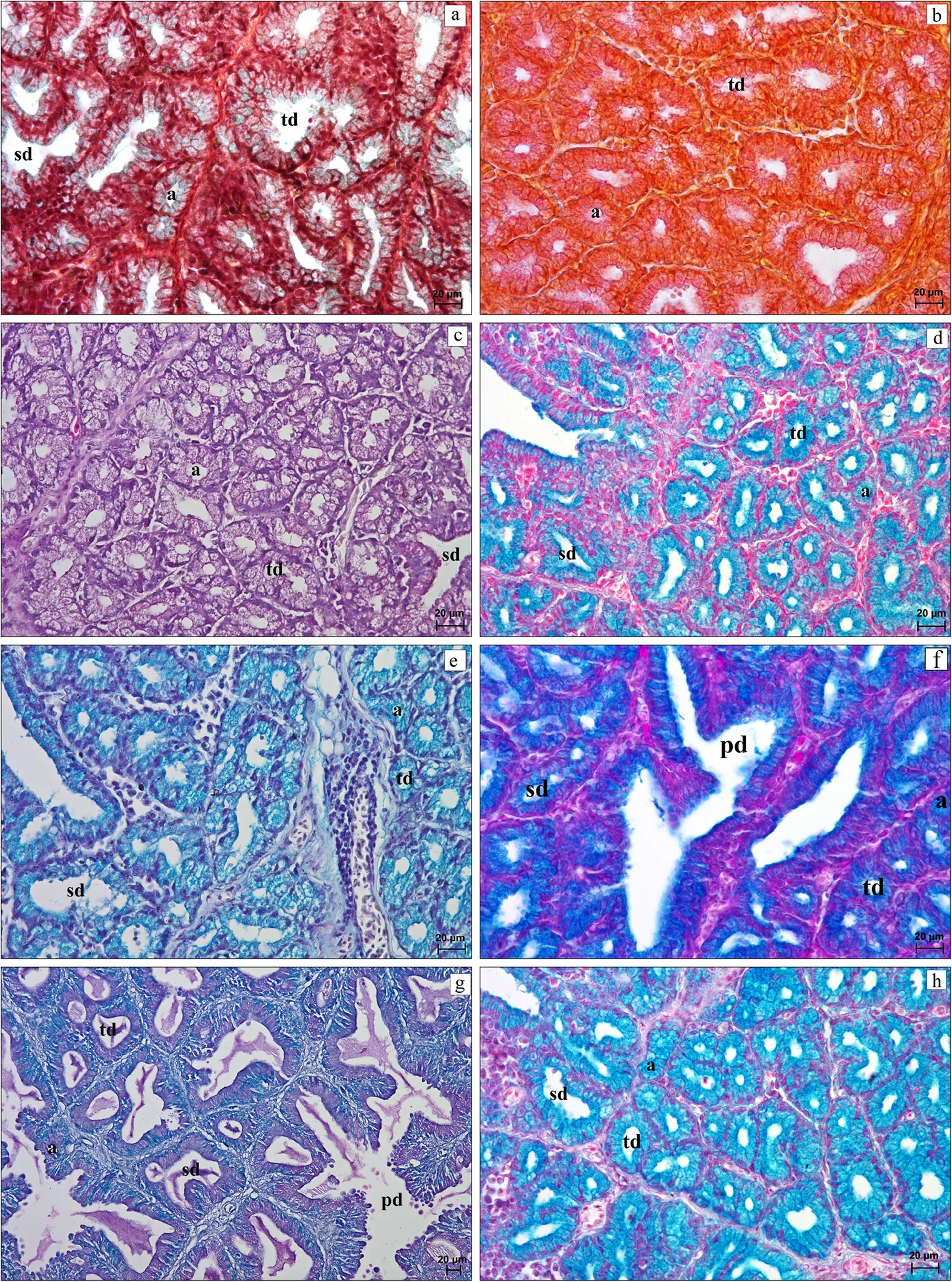
Histochemical profile of the Harderian gland in the Great Spotted Woodpecker. Representative micrographs from adult and subadult specimens show the distribution of histochemical reactions within the secretory acini and duct system. Movat pentachrome illustrates the general arrangement of acini and ducts within the glandular parenchyma. Mucicarmine, PAS, Alcian blue, AB/PAS, paraldehyde fuchsin, and colloidal iron staining demonstrate the distribution of glycoconjugates and mucosubstances in the secretory acini and ductal epithelium. PAS staining shows no clear neutral component, whereas Alcian blue, AB/PAS, paraldehyde fuchsin, and colloidal iron staining highlight acidic mucosubstances, particularly within the secretory portions and ductal system. These reactions indicate that the Harderian gland of the Great Spotted Woodpecker has a distinct histochemical secretory profile involving both acinar and ductal compartments. Abbreviations: a, acini; pd, primary duct; sd, secondary duct; td, tertiary duct. (**a**) Movat pentachrome (modified Russell-Movat); (**b**) mucicarmine; (**c**) periodic acid–Schiff; (**d**) Alcian blue at pH 1.0; (**e**) Alcian blue at pH 2.5; (**f**) AB pH 2.5/PAS; (**g**) paraldehyde fuchsin, Gomori method; (**h**) colloidal iron. Scale bars = 20 µm.

Histochemical staining revealed regional differences in the distribution and intensity of mucosubstances within the secretory units and duct system of the Harderian gland. Movat pentachrome staining showed a weak reaction, assessed as −/+ (Fig. 5a). Mucicarmine staining showed a weak positive reaction (+), with pale to moderate pink staining in selected secretory areas (Fig. 5b). The PAS reaction was negative in the secretory epithelium and ductal system, suggesting an absence or very low amount of PAS-positive neutral mucins and glycoproteins in the examined sections (Fig. 5c). Alcian blue at pH 1.0 showed a moderate positive reaction (++), observed in the secretory acini and in the primary, secondary, and tertiary ducts. This staining pattern was consistent with the presence of strongly sulfated acidic mucosubstances (Fig. 5d). The strongest reactions were observed with Alcian blue at pH 2.5 and colloidal iron, both in the secretory acini and throughout the duct system. Alcian blue at pH 2.5 showed a strong positive reaction (+++), indicating abundant acidic mucosubstances, including carboxylated and/or weakly sulfated glycoconjugates (Figs. 5e, h). The combined AB pH 2.5/PAS method produced a strong blue reaction (+++) in the secretory acini and duct system, whereas no clear magenta PAS-positive component was observed (Fig. 5f). This pattern indicated a predominance of acidic mucosubstances. Colloidal iron staining showed a strong positive reaction (+++) in the secretory acini and duct system, consistent with the presence of sulfated and carboxylated acidic mucosubstances. Paraldehyde fuchsin staining produced a violet reaction, assessed as +/++, in the secretory acini and at all levels of the duct system, which was interpreted as evidence of sulfated acidic mucins (Fig. 5g). Toluidine blue staining showed a strong metachromatic reaction in selected secretory epithelial cells and in the ductal contents, also consistent with acidic mucosubstances in the Harderian gland (Fig. 4e).

### Histological and histochemical characteristics of the lacrimal gland

**Fig. 6:**
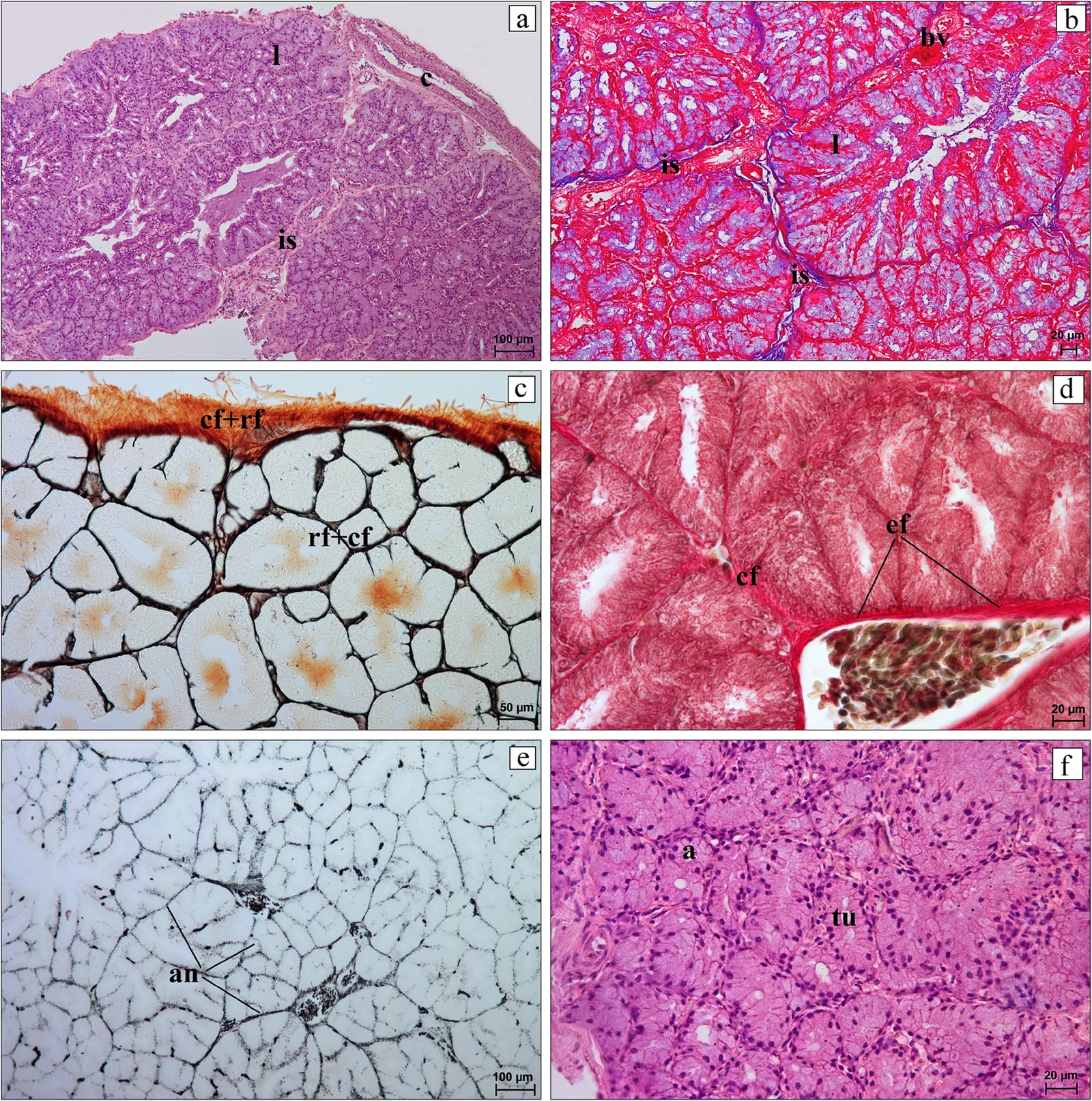
Histological organization of the lacrimal gland in the Great Spotted Woodpecker. Representative micrographs from well-preserved adult and subadult specimens show a lobulated gland surrounded by a connective tissue capsule. Interlobular septa extend from the capsule into the glandular parenchyma and contain blood vessels, connective tissue fibers, and nerve fibers/axonal elements. Hematoxylin and eosin staining shows the general lobular arrangement of the gland and the distribution of tubuloacinar secretory units within the lobules. Heidenhain’s Azan trichrome highlights collagen-rich connective tissue in the capsule and interlobular septa, whereas reticulin staining demonstrates the delicate reticular fiber network supporting the glandular parenchyma. Verhoeff staining reveals elastic fibers within the stromal connective tissue, and Bielschowsky staining demonstrates axonal elements/nerve fibers associated with the glandular stroma. Abbreviations: a, acini; an, axonal elements/nerve fibers; bv, blood vessel; c, capsule; cf, collagen fibers; ef, elastic fibers; is, interlobular septum; l, lobule; rf, reticular fibers; tu, tubuloacinar secretory units. (**a**, **f**) Mayer’s hematoxylin and eosin; (**b**) Heidenhain’s Azan trichrome; (**c**) reticulin; (**d**) Verhoeff; (**e**) Bielschowsky. Scale bars: (**a**, **e**) 100 µm; (**b**, **d**, **f**) 20 µm; (**c**) 50 µm

**Fig. 7:**
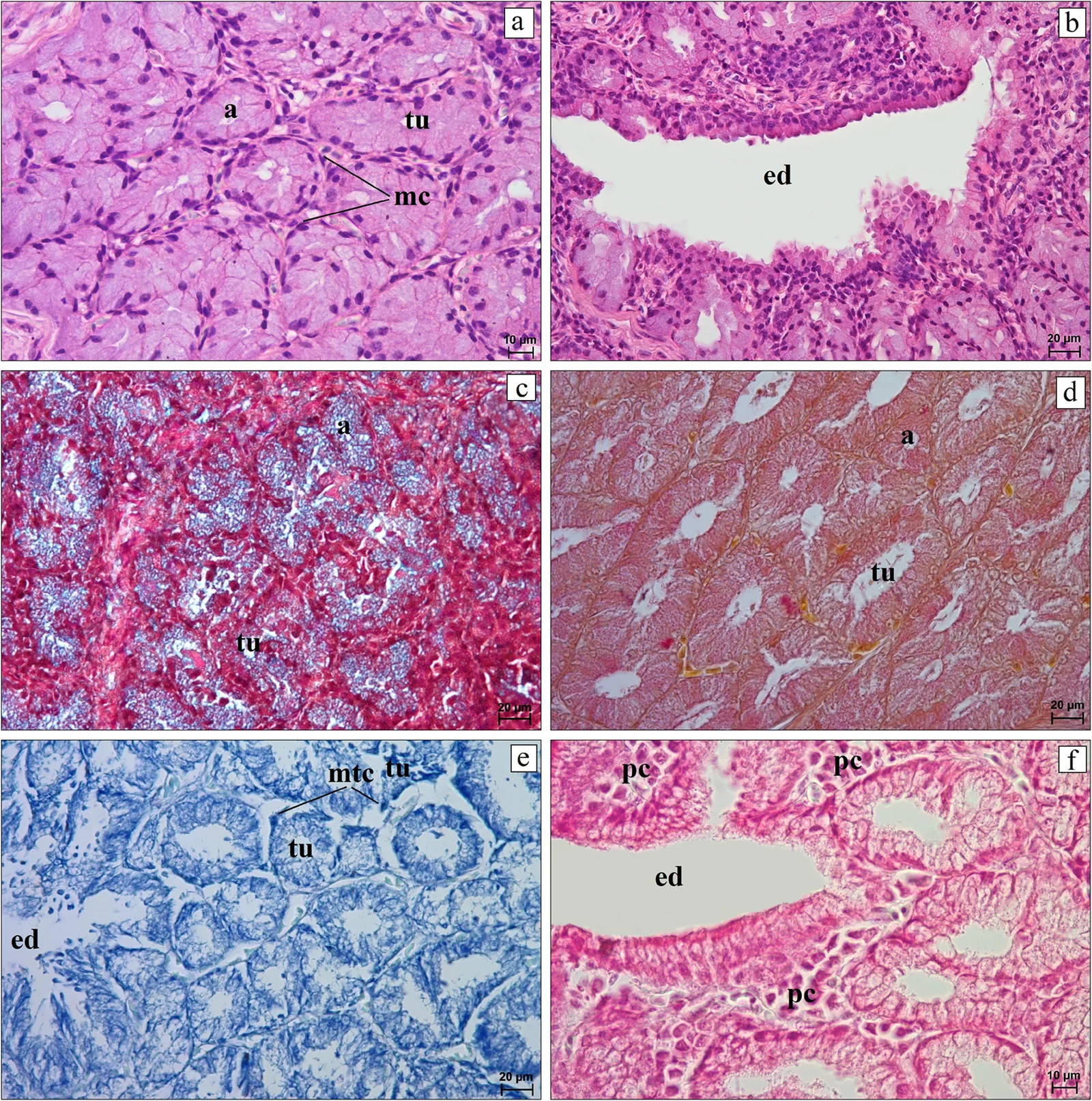
Secretory and ductal components of the lacrimal gland in the Great Spotted Woodpecker. Representative micrographs from adult and subadult specimens show closely arranged acini and tubuloacinar secretory units associated with excretory ducts. Hematoxylin and eosin staining demonstrates the general arrangement of the secretory portions and ductal profiles, with flattened myoepithelial cells visible along the basal surface of selected acini and ducts. Movat pentachrome staining shows the connective tissue framework surrounding the secretory and ductal compartments. Mucicarmine staining highlights mucosubstance-containing elements within the glandular parenchyma. Toluidine blue polychrome staining demonstrates metachromatic mast cells within the stromal connective tissue, whereas methyl green–pyronin staining shows periductal plasma cells, particularly around the excretory ducts. Abbreviations: a, acini; ed, excretory duct; mc, myoepithelial cells; mtc, metachromatic mast cells; pc, plasma cells; tu, tubuloacinar secretory units. (**a**, **b**) Mayer’s hematoxylin and eosin; (**c**) Movat pentachrome (modified Russell-Movat); (**d**) mucicarmine; (**e**) toluidine blue polychrome; (**f**) methyl green–pyronin. Scale bars: (**a**, **f**) 10 µm; (**b**–**e**) 20 µm

The lacrimal gland was enclosed by a thick connective tissue capsule. Thick connective tissue septa extended from the capsule into the glandular parenchyma and divided it into lobes of variable size (Figs. 6a, b). The capsule and septa contained abundant collagen and reticular fibers, sparse elastic fibers, axonal structures, and numerous large blood vessels (Figs.6c–e). Histologically, the lacrimal gland showed a multilobar, compound tubuloacinar organization (Fig. 6f). The secretory acini were surrounded by myoepithelial cells and were composed of tall conical epithelial cells forming narrow lumina (Fig. 7a). The nuclei of the acinar cells were oval and located in the basal part of the cells. The cytoplasm was granular and eosinophilic (Fig. 7a). The tubular secretory units were lined by cuboidal epithelial cells (Fig. 7a). Large, branched excretory ducts with wide lumina were visible in the central part of each lobe (Fig. 7b). These ducts were lined by simple columnar epithelium (Fig. 7b). The lacrimal gland in the examined woodpeckers showed features consistent with mucous secretion (Figs. 7c, d). Mucicarmine staining showed a moderate positive reaction (++), with pink staining in the secretory epithelium and secretory material. Movat pentachrome staining showed a moderate to strong reaction (++/+++), mainly within the secretory units and ductal system (Figs. 7c, d). Toluidine blue staining showed a weak metachromatic reaction in selected secretory areas and in the ductal contents. Metachromatic mast cells were occasionally observed in the connective tissue septa, mainly close to blood vessels and excretory ducts (Fig. 7e). Organized lymphoid aggregates were not observed in the examined sections, whereas numerous plasma cells were present in the connective tissue surrounding the excretory ducts (Fig. 7f).

**Fig. 8:**
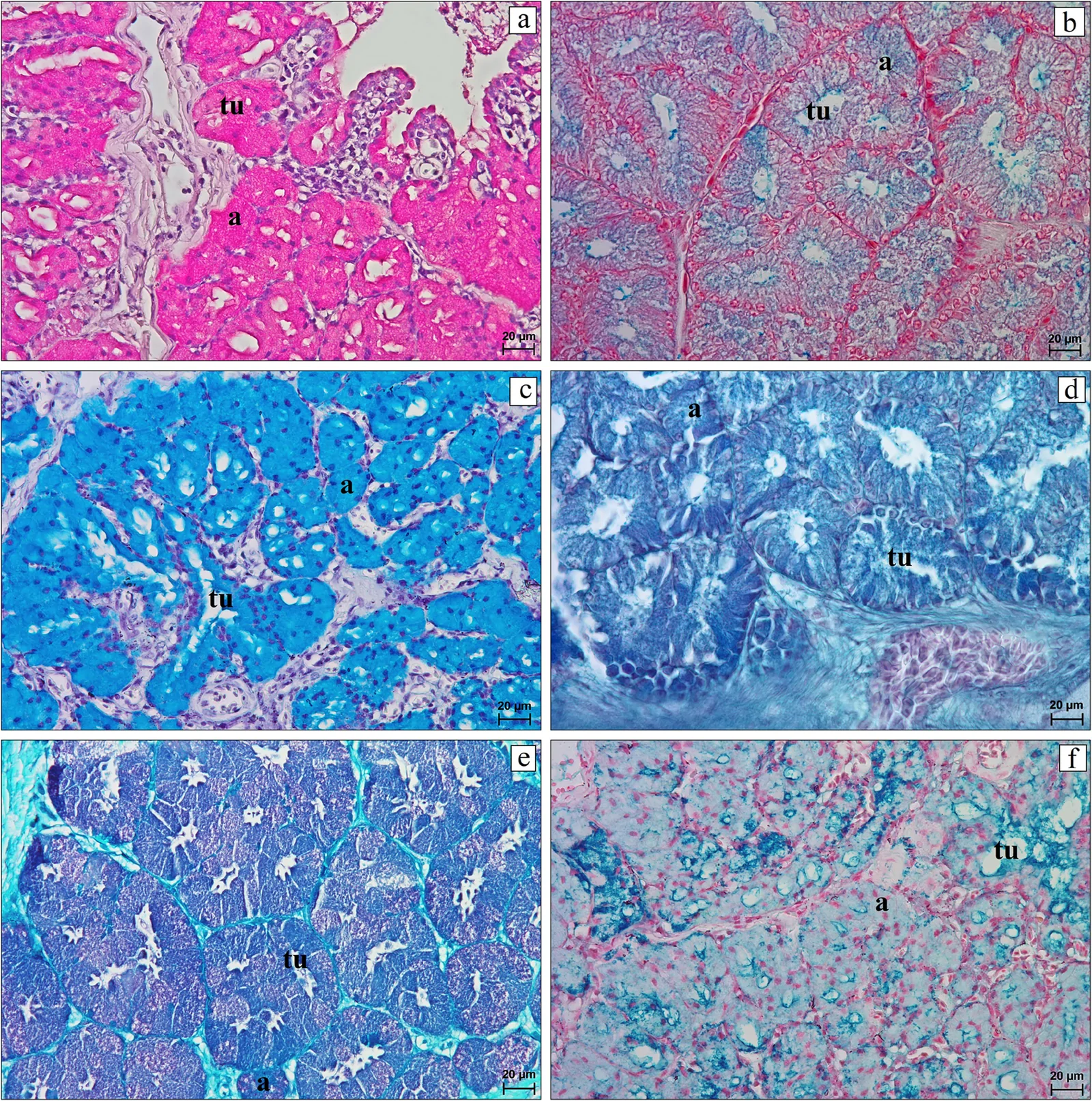
Histochemical profile of the lacrimal gland in the Great Spotted Woodpecker. Representative micrographs from adult and subadult specimens show histochemical staining reactions within the acini and tubuloacinar secretory units. PAS staining demonstrates neutral glycoconjugates in the secretory epithelium and luminal material, whereas Alcian blue at pH 1.0 and pH 2.5 highlights acidic mucosubstances within the glandular secretory portions. AB pH 2.5/PAS staining shows mixed neutral and acidic components, indicating a heterogeneous secretory profile of the lacrimal gland. Paraldehyde fuchsin, Gomori method, and colloidal iron staining further demonstrate acidic mucosubstances in the acini and tubuloacinar units. Abbreviations: a, acini; tu, tubuloacinar secretory units. (**a**) periodic acid–Schiff; (**b**) Alcian blue at pH 1.0; (**c**) Alcian blue at pH 2.5; (**d**) AB pH 2.5/PAS; (**e**) paraldehyde fuchsin, Gomori method; (**f**) colloidal iron. Scale bars = 20 µm

Histochemical staining demonstrated pronounced reactions for both neutral and acidic mucosubstances in the lacrimal gland. The PAS reaction was strongly positive (+++), showing abundant PAS-positive glycans, glycoproteins, and neutral mucins in the glandular epithelium and secretory material (Fig.8a). Alcian blue at pH 1.0 showed a weak positive reaction (+), suggesting a low amount of strongly sulfated acidic mucosubstances (Fig. 8b). In contrast, Alcian blue at pH 2.5 showed a strong positive reaction (+++) in the secretory units and ducts, consistent with abundant acidic mucosubstances, including carboxylated and/or weakly sulfated glycoconjugates (Fig. 8c). The combined AB pH 2.5/PAS staining showed a predominantly blue reaction, assessed as ++/+++, in the acini and tubules (Fig. 8d). This staining pattern suggested a predominance of acidic mucosubstances over neutral PAS-positive components in the examined sections. Paraldehyde fuchsin staining showed a strong positive reaction (+++), which was interpreted as evidence of abundant sulfated acidic mucins (Fig. 8e). Colloidal iron staining showed a moderate to strong positive reaction (++/+++), supporting the presence of sulfated and carboxylated acidic mucosubstances (Fig. 8f). Toluidine blue staining showed a weak metachromatic reaction (+), suggesting a limited amount of metachromatic acidic mucosubstances in the examined glandular tissue (Fig. 7e).

## Discussion

This study describes the topography, macroscopic morphology, histological organization, and histochemical profile of the Harderian and lacrimal glands in the Great Spotted Woodpecker. These glands occupied distinct orbital regions: the Harderian gland was associated with the ventromedial part of the orbit and the nictitating membrane apparatus, whereas the lacrimal gland occupied a dorsolateral position. This arrangement agrees with the general organization of avian orbital glands (Wight et al. 1971a; Burns 1976, 1992; Burns and Maxwell 1979; Payne 1994; Shirama et al. 1996; Jones et al. 2007; Kern 2007; Harris et al. 2008; Klećkowska-Nawrot et al. 2015b, 2016a).

The single excretory duct of the Harderian gland opened into the lower conjunctival sac, between the nictitating membrane and the cornea. This position is consistent with a contribution of Harderian gland secretion to lubrication and protection of the ocular surface and nictitating membrane. However, this interpretation remains morphological only, because secretory activity and tear film composition were not evaluated in vivo. The close relationship among the Harderian gland, nictitating membrane, and ocular surface has also been emphasized in other avian species in both secretory and immunological contexts (Burns 1992; Shirama et al. 1996).

Histologically, the Harderian gland in the examined material showed a Type I compound tubuloacinar organization, with lobules of variable size and a distinct central ductal system. Similar connective tissue organization, including a capsule, interlobular septa, blood vessels, and nerve fibers, has been reported in several birds, including chickens, geese, ostriches, capercaillies, pheasants, and selected free-ranging species (Boydak and Aydin 2009; Mobini 2012; Klećkowska-Nawrot et al. 2015b, 2016a, b; Beheiry et al. 2020). In the Great Spotted Woodpecker, the primary ducts were large, irregular, and often branched, with secondary and tertiary ducts extending toward the peripheral secretory acini. This arrangement corresponds to the central ductal organization described as a characteristic feature of the avian Harderian gland (Wight et al. 1971a; Burns and Maxwell 1979; Boydak and Aydin 2009; Beheiry et al. 2020).

The secretory component of the Harderian gland was relatively uniform and consisted of one main type of secretory unit and one predominant epithelial cell type. This contrasts with some avian species in which a more heterogeneous epithelial organization has been described. For example, two epithelial cell types were reported in the glandular tubules of the Bilgoraj goose, and structural differences were observed between the common pheasant and hybrids of the Italian amber pheasant and common pheasant (Klećkowska-Nawrot et al. 2015b, 2016a). Compared with these species, the Harderian gland of the Great Spotted Woodpecker appears to have a more homogeneous secretory parenchyma combined with a well-developed duct system.

A notable feature of the Harderian gland was the presence of plasma cells and lymphocytes, especially around the primary ducts. In birds, the Harderian gland is regarded not only as a secretory organ but also as a component of local mucosal defense associated with the ocular surface and upper respiratory tract. Plasma cells, Russell bodies, and immunoglobulins have been described in the Harderian gland of the domestic fowl (Wight et al. 1971a, b; Burns 1992; Scott et al. 1993; Shirama et al. 1996; Khan et al. 2007). Scott et al. (1993) showed that plasma cells in the chicken Harderian gland may produce IgA, IgG, and IgM, and that these immunoglobulins may contribute to ocular surface protection. In the Great Spotted Woodpecker, plasma cells did not form extensive lymphoid aggregates but were concentrated mainly in the periductal region. Thus, the lymphoid component in the examined material had a predominantly diffuse and periductal pattern.

The histochemical profile of the Harderian gland was dominated by acidic mucosubstances. The strongest reactions were observed with Alcian blue pH 2.5, the combined AB pH 2.5/PAS method, and colloidal iron, whereas the PAS reaction was negative in the secretory epithelium and ductal system. This differs from many avian species in which both acidic and neutral glycoconjugates have been reported in the Harderian gland, with variable proportions depending on species and glandular region (Wight et al. 1971a, b; Brobby 1972; Boydak and Aydin 2009; Mobini 2012; Klećkowska-Nawrot et al. 2015b, 2016b; Beheiry et al. 2020). In the Great Spotted Woodpecker, the absence of a clear PAS-positive component, together with strong reactions for acidic mucosubstances, suggests a predominance of acidic secretory components in the examined sections.

The positive Alcian blue pH 1.0 and paraldehyde fuchsin reactions further suggest the presence of sulfated acidic mucins. Such components may increase the viscosity and protective properties of glandular secretion and may support maintenance of the ocular surface and nictitating membrane. Similar roles have been proposed for Harderian gland mucosubstances in other birds, where they have been associated with moistening, lubrication, and mechanical protection of the ocular surface (Wight et al. 1971b; Burns 1992; Payne 1994; Beheiry et al. 2020). In the Great Spotted Woodpecker, this secretory profile may be functionally relevant because the head and orbital region may be exposed to bark particles, wood dust, and small organic debris during foraging and pecking. This interpretation, however, remains indirect and requires confirmation by studies of tear film composition and glandular secretion.

The lacrimal gland differed from the Harderian gland in both topography and structure. It was elongated, flattened, and macroscopically undivided, and it drained by multiple excretory ducts into the dorsal conjunctival fornix. Similar dorsolateral positioning and contribution of the lacrimal gland to the ocular surface have been described in other birds (Burns 1976; Jones et al. 2007; Kern 2007; Harris et al. 2008; Klećkowska-Nawrot et al. 2015c, 2016b). However, the shape and arrangement of the lacrimal gland vary among species. For example, a flattened gland was described in the capercaillie, whereas in the little owl the gland was cylindrical and located along the dorsal orbital margin (Klećkowska-Nawrot et al. 2016b; Shawki et al. 2019). These comparisons indicate that, despite a broadly conserved topographic pattern, the avian lacrimal gland may show species-related variation in shape, adjacent relationships, and drainage pattern.

Microscopically, the lacrimal gland showed a multilobar, compound tubuloacinar organization and a well-developed connective tissue framework. Its thick capsule and interlobar septa were more pronounced than those of the Harderian gland. Similar lobular or multilobar organization has been reported in other birds, including the ostrich, capercaillie, and little owl (Klećkowska-Nawrot et al. 2015c, 2016b; Shawki et al. 2019). The large, branched excretory ducts in the central parts of the lobes may facilitate secretion drainage into the conjunctival sac, although this functional interpretation would require physiological confirmation.

Numerous plasma cells were observed in the connective tissue surrounding the lacrimal gland ducts, whereas organized lymphoid aggregates were not found. This pattern differs partly from that reported in the capercaillie, in which lymphocytes and plasma cells were not observed in the lacrimal gland, although the Harderian gland contained numerous lymphoid cells (Klećkowska-Nawrot et al. 2016b). In the Great Spotted Woodpecker, the immune component of the lacrimal gland therefore had a diffuse and periductal distribution. This pattern supports, but does not prove, a role of the lacrimal gland in local ocular surface defense. Immunohistochemical studies would be needed to characterize these cells more precisely.

The lacrimal gland showed a mixed histochemical profile, with both neutral and acidic mucosubstances. The strong PAS reaction indicated abundant PAS-positive glycans, glycoproteins, and neutral mucins, whereas the strong Alcian blue pH 2.5 and colloidal iron reactions indicated a considerable amount of acidic mucosubstances, including carboxylated and/or weakly sulfated glycoconjugates. In the ostrich, PAS and Alcian blue pH 2.5 reactivity varied with age, whereas in the capercaillie these reactions were weak in the lacrimal gland (Klećkowska-Nawrot et al. 2015c, 2016b). Compared with these species, the lacrimal gland of the Great Spotted Woodpecker was characterized by both a strong PAS-positive component and a pronounced acidic component.

The combined AB pH 2.5/PAS method showed a predominantly blue reaction, despite the strong PAS reaction observed in separate staining. This pattern suggests that acidic mucosubstances were particularly prominent in the examined secretory units and ducts. The coexistence of neutral and acidic mucosubstances has also been described in the orbital glands of other birds, including ducks, capercaillies, little owls, hoopoes, and cattle egrets, although the proportions of these components differ among species (Dimitrov and Nikiforov 2005; Klećkowska-Nawrot et al. 2016b; Shawki et al. 2019; Al-Nefeiy et al. 2022). The strong paraldehyde fuchsin reaction further indicates a marked contribution of sulfated acidic mucins to the secretory profile of the lacrimal gland.

Taken together, the Harderian and lacrimal glands differed clearly in their histochemical profiles. The Harderian gland was dominated by acidic mucosubstances and lacked a distinct PAS-positive component, whereas the lacrimal gland contained both PAS-positive components and abundant acidic mucosubstances, including sulfated mucins. This difference suggests partial functional differentiation between the two glands. The Harderian gland may contribute mainly acidic protective components, whereas the lacrimal gland may provide both glycoprotein-rich and acidic components to the precorneal tear film. Similar differences between the Harderian and lacrimal glands have been reported in other birds, including the capercaillie and ostrich (Klećkowska-Nawrot et al. 2015c, 2016b).

No consistent sex-related differences were apparent in the general histological organization or histochemical profile of either gland. This observation should be interpreted descriptively, because the study was not designed to statistically evaluate sex-related variation. Similar observations regarding the absence of clear sex-related differences in Harderian gland structure have been reported in some previous avian studies (Oliveira et al. 2006; Khan et al. 2007; Boydak and Aydin 2009; Mobini 2012; Beheiry et al. 2020). In the present study, the main objective was to characterize the morphology and histochemistry of the orbital glands in the examined microscopic material.

The juvenile Harderian and lacrimal gland samples showed early postmortem autolytic changes; therefore, age-related variation could not be assessed. Because of the descriptive nature of the study and the limited number of nonjuvenile specimens, sex-related differences were not evaluated statistically.

In summary, the Harderian and lacrimal glands of the Great Spotted Woodpecker follow the basic structural plan typical of avian orbital glands, but they differ in topography, ductal organization, connective tissue framework, plasma cell distribution, and histochemical profile. These differences suggest complementary contributions to ocular surface maintenance and local mucosal protection. From a veterinary and comparative anatomical perspective, these findings provide baseline information for recognizing the normal structure of the lacrimal apparatus in a free-ranging avian species.

## Conclusions

This study provides the first detailed description of the Harderian and lacrimal glands in the Great Spotted Woodpecker. Both glands were compound and tubuloacinar but differed in topography, ductal arrangement, connective tissue framework, plasma cell distribution, and histochemical profile. Harderian gland secretion was dominated by acidic mucosubstances, whereas the lacrimal gland showed a mixed secretory profile. Macroscopic morphometry showed that the Harderian gland had a higher size index than the lacrimal gland.

The histochemical profiles of both glands are consistent with their contribution to lubrication and protection of the ocular surface. The presence of plasma cells, especially around the ductal system, supports a possible role in local mucosal defense, although this requires confirmation by immunohistochemical methods.

## Supplementary Information

Below is the link to the electronic supplementary material.

Supplementary Material 1

Supplementary Figure 9 (PNG 548 KB)

High Resolution Image (TIF 3.31 MB)

Supplementary Material 3

## Data Availability

No datasets were generated or analysed during the current study.
