# Supplementary material for: Anatomy, histology, and histochemistry of the harderian and lacrimal glands in the great spotted woodpecker (dendrocopos major)
Source: Vet Res Commun. 2026 Sep 16;50(6):574. doi: 10.1007/s11259-026-11477-w (PMC13582243; doi:10.1007/s11259-026-11477-w)
Supplement: Supplementary file 1 [file 11259_2026_11477_MOESM1_ESM.docx]

**Figure S1.**

The results of amplification of intron 16 of the CHD1 gene used as a molecular marker for sex determination in eighteen Great Spotted Woodpecker individuals (lanes 1–18; see Table 1). Lane DL - GeneRuler 100 bp Plus DNA Ladder (Thermo Scientific).
