# Supplementary material for: Anatomy, histology, and histochemistry of the harderian and lacrimal glands in the great spotted woodpecker (dendrocopos major)
Source: Vet Res Commun. 2026 Sep 16;50(6):574. doi: 10.1007/s11259-026-11477-w (PMC13582243; doi:10.1007/s11259-026-11477-w)
Supplement: Supplementary file 2 [file 11259_2026_11477_MOESM3_ESM.docx]

**Table S1**. Detailed description of histological and histochemical stainings and the scientific outcome.

| **No.** | **Staining** | **Structure** | **Manufacturer** |
| --- | --- | --- | --- |
| 1. | Mayer’s hematoxylin and eosin | general histological assessment of the glandular parenchyma, ducts, capsule, interlobular connective tissue, blood vessels, and surrounding tissues | Sigma-Aldrich St. Louis, MO, USA; cat. no. MHS16 |
| 2. | Azan trichrome | collagen fibers and connective tissue organization | Bio-Optica Milano S.p.A., Milan, Italy; cat. no. 04-001802 |
| 3. | Mallory trichrome with aniline blue | collagen fibers and connective tissue organization | Bio-Optica Milano S.p.A.; cat. no. 04-020802 |
| 4. | Reticulin | reticular fibers visualization | BioGnost Ltd., Zagreb, Croatia; cat. no. RE-100T |
| 5. | Verhoeff | elastic fibers detection | BioGnost Ltd.; cat. no. VER-100T |
| 6. | Bielschowsky | axons and neurofibrillar structures | BioGnost Ltd.; cat. no. BLS-100T |
| 7. | Toluidine blue polychrome | metachromatic components identification, mainly mast cell granules and acidic mucosubstances, in the glandular interstitium and around the ductal system | Bio-Optica Milano S.p.A.; cat. no. 05-B23001 |
| 8. | Methyl green–pyronin | identification of plasma cells, which showed pink-red cytoplasmic staining | Bio-Optica Milano S.p.A.; cat. no. 05-B15003 |
| 9. | Movat pentachrome, modified Russell–Movat method | acidic mucopolysaccharides and glycosaminoglycans detection | BioGnost Ltd.; cat. no. MOV-100T |
| 10. | Mucicarmine | acid mucins (pink to red color) and neutral mucins (light yellow color) identification | Bio-Optica Milano S.p.A.; cat. no. 04-190812 |
| 11. | Periodic acid–Schiff reaction | glycans, glycoproteins, and neutral mucins demonstration | PAS; Bio-Optica Milano S.p.A.; cat. no. 04-130802 |
| 12. | Alcian blue at pH 1.0 | strongly sulfated glycosaminoglycans identification | AB pH 1.0; Bio-Optica Milano S.p.A.; cat. no. 04-166802 |
| 13. | Alcian blue at pH 2.5 | acidic mucosubstances, including carboxylated and weakly sulfated glycosaminoglycans detection | AB pH 2.5; Bio-Optica Milano S.p.A.; cat. no. 04-160802 |
| 14. | Combined AB pH 2.5/PAS method | used to distinguish acidic mucosubstances, stained blue, from neutral mucins, stained magenta | Bio-Optica Milano S.p.A.; cat. no. 04-163802 |
| 15. | Paraldehyde fuchsin, Gomori method | sulfated acidic mucins identification | Bio-Optica Milano S.p.A.; cat. no. 04-D45872 |
| 16. | Colloidal iron method | sulfated and carboxylated acidic mucosubstances detection | Bio-Optica Milano S.p.A.; cat. no. 04-180809 |
